# Loss of Merlin induces metabolomic adaptation that engages dependence on Hedgehog signaling

**DOI:** 10.1038/srep40773

**Published:** 2017-01-23

**Authors:** Shamik Das, William P. Jackson, Jeevan K. Prasain, Ann Hanna, Sarah K. Bailey, J. Allan Tucker, Sejong Bae, Landon S. Wilson, Rajeev S. Samant, Stephen Barnes, Lalita A. Shevde

**Affiliations:** 1 Department of Pathology, University of Alabama at Birmingham, Birmingham, AL 35233, USA; 2 Department of Pharmacology and Toxicology, University of Alabama at Birmingham, Birmingham, AL 35233, USA; 3 University of South Alabama, AL 36617, United States; 4 Comprehensive Cancer Center, University of Alabama at Birmingham, Birmingham, AL 35233, USA; 5 Department of Medicine, University of Alabama at Birmingham, Birmingham, AL 35233, USA; 6 Targeted Metabolomics and Proteomics Laboratory, University of Alabama at Birmingham, Birmingham, AL 35233, USA.

## Abstract

The tumor suppressor protein Merlin is proteasomally degraded in breast cancer. We undertook an untargeted metabolomics approach to discern the global metabolomics profile impacted by Merlin in breast cancer cells. We discerned specific changes in glutathione metabolites that uncovered novel facets of Merlin in impacting the cancer cell metabolome. Concordantly, Merlin loss increased oxidative stress causing aberrant activation of Hedgehog signaling. Abrogation of GLI-mediated transcription activity compromised the aggressive phenotype of Merlin-deficient cells indicating a clear dependence of cells on Hedgehog signaling. In breast tumor tissues, GLI1 expression enhanced tissue identification and discriminatory power of Merlin, cumulatively presenting a powerful substantiation of the relationship between these two proteins. We have uncovered, for the first time, details of the tumor cell metabolomic portrait modulated by Merlin, leading to activation of Hedgehog signaling. Importantly, inhibition of Hedgehog signaling offers an avenue to target the vulnerability of tumor cells with loss of Merlin.

The *NF2* gene encodes the tumor suppressor protein Merlin. Originally described as mutated in the context of neurofibromatosis and tumors of the nervous system, Merlin has more recently been demonstrated to function as a tumor suppressor in mesothelioma, melanoma, and breast cancer^1^,^2^,^3^,^4^,^5^,^6^. Multiple post-translational modifications and interactions with cytoskeletal proteins regulate and execute Merlin’s tumor suppressor function. Mutations in the *NF2* gene are rare in breast cancer; instead, Merlin protein levels decrease with disease progression and metastasis due to post-translational degradation^1^.

The molecular functions of Merlin have been described at the cortical membrane and in the nucleus. Merlin binds and interacts with the actin and microtubule cytoskeleton^7^,^8^,^9^. Merlin associates with α-catenin as an essential component of the formation of adherens junctions, exerts contact-dependent inhibition of proliferation by interacting with CD44 in response to hyaluronan, sequesters EGFR from signaling stimuli, and directly inhibits p21-activating kinase (PAK)^10^,^11^,^12^,^13^,^14^,^15^. Functionally, Merlin inhibits contact-dependent growth and suppresses invasiveness of tumor cells. In animal models, Merlin has been demonstrated to suppress xenograft formation of multiple cancer types. Loss of Merlin enables unrestricted growth of tumor cells characterized by the aberrant upregulation of several growth-promoting signaling events in the tumor cells^16^.

The process of tumorigenesis and tumor progression is associated with metabolic changes that enable tumor cells to evolve and progress. Recent advances have defined perspectives of metabolomic profiling of cancer that have defined treatment response, ‘stemness’, oxidative stress, changes with respect to the estrogen receptor (ER) status of breast cancer etc.^17^,^18^,^19^. Our work on the loss of Merlin has revealed a vital role for Merlin in advanced breast cancer^1^,^20^. We adopted an untargeted approach to investigate metabolomic changes impacted in breast cancer cells by Merlin. Based on MS/MS fragmentation data, we discerned novel, specific changes in glutathione metabolites associated with Merlin. Following these leads, we determined that loss of Merlin increases oxidative stress which causally leads to aberrant activation of Hedgehog (Hh) signaling. In this work we, for the first time, show that Merlin alters the global cancer cell metabolome to interface with the Hh pathway leading to the activation of an invasive cellular program. We present that, in breast cancer where there is loss of Merlin, Hh inhibitors have the potential to open a new yet uncharted vista for therapeutics.

## Results

### Merlin alters distinct sets of glutathione-derived metabolites in breast cancer cells

**Figure 1:**
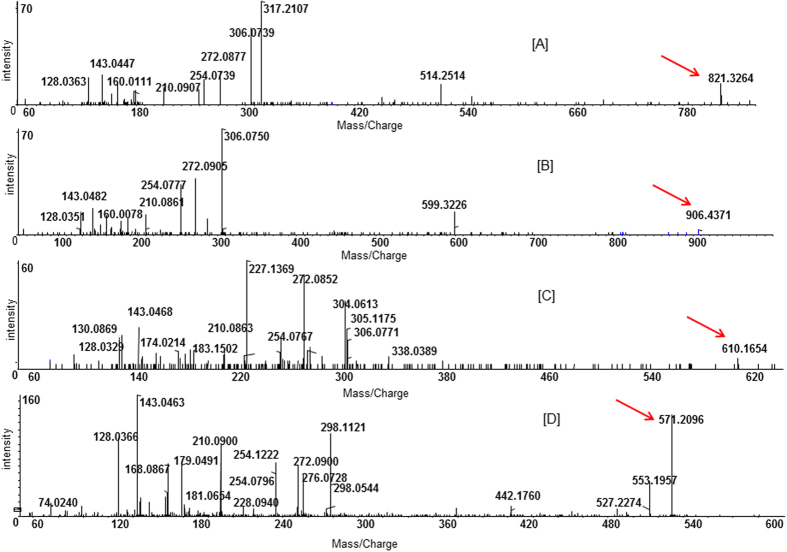
Negative ion product ions spectra of GSH containing metabolites. The proposed GSH conjugates were structurally divided into three classes: *Thioester GSH conjugates:* Three thioester class GSH metabolites with *m/z* 821.3264, 906.4371 and 468.1325 were observed. (**A**) The ESI-MS/MS spectrum of *m/z* 821.3264 precursor ion. The product ion spectrum of this ion showed a neutral loss of 307 Da and prominent product ion fragments at *m/z* 306.0739 as *m/z* 272.0877, 254.0739, 210.0907, 179.0504, 160.0111, 143.0447 and 128.0363. This fragmentation pattern is very similar to that of the glutathionyl-derived species observed in the negative ion mode^46^. The presence of product ions *m/z* 306.0739 and 272.0877 together indicated this metabolite likely to be a thioester GSH conjugate. (**B**) The MS/M:S spectrum of *m/z* 906.4371. The presence of a product ion *m/z* 306.0761 after a neutral loss of 162.0 Da indicated the GSH moiety is likely to be conjugated with a hexose sugar. *Disulfide GSH conjugates:* We observed two disulfide GSH conjugates (*m/z* 610.1654 and 644.1570) of unknown structures. (**C**) The product ion spectrum of *m/z* 610.1654. A series of product ions corresponding to glutathionyl-derived species were observed. Similarly, MS/MS of *m/z* 644.1570 generated intense product ions with *m/z* 339.1021, 306.0750, 338.0378, 306.0750 and 304.0614. The ions *m/z* 306.0750 and 338.0378 represent deprotonated tripeptide persulfides. Based on these mass spectrometric data, both metabolites (*m/z* 610.1654 and 644.1570) were identified as disulfide GSH conjugates. (**D**) MS/MS spectra *m/z* 571.2096. *Benzylic GSH conjugate:* The [M-H]^−^ precursor ion *m/z* 571.2096, upon MS/MS fragmentation, produced ions *m/z* 128.0366, 143.0463, 179.0491, 210.0900, 254.0796 and 272.0900, representing the glutathionyl moiety. The absence of product ion *m/z* 306 in the spectrum indicated that the product ions are generated exclusively from the cleavage of bonds within the gluthathionyl moiety^47^. The loss of 129 Da can be rationalized by the elimination of a neutral pyroglutamic acid moiety giving rise to *m/z* 442.1760. These pieces of information indicate that this metabolite is likely a benzylic GSH conjugate.

To elucidate the impact of altered Merlin levels, we evaluated two isogenic systems of breast cancer cells - SUM159 metastatic breast cancer cells and MCF10AT non-metastatic breast cancer cells. SUM159 cells (do not express detectable endogenous Merlin protein) were restored for Merlin while MCF10AT cells that express appreciable levels of Merlin were engineered to stably knockdown Merlin (Supplementary Figure 1B). Cell lysates were analyzed for intracellular metabolites by nano-LC-MS/MS on a SCIEX 5600 triple-TOF (Supplementary Figure 2). Overall 47 metabolites were significantly (p < 0.05) altered in the context of Merlin. Most notably, *a*mong the 30 unreported metabolites detected in negative ion mode, six metabolites (20%) were identified as GSH conjugates belonging to thioesters; disulfides; benzylic classes on the basis of interpretation of their product ions and comparison to reference data (Fig. 1A–D; Supplementary Figures 3 and 4; Supplementary Tables 1–6), collectively indicating that specific to the loss of Merlin is a change in multiple glutathione derivatives.

### Loss of Merlin induces reactive oxygen species (ROS) concomitant with lower levels of reduced glutathione (GSH)

**Figure 2:**
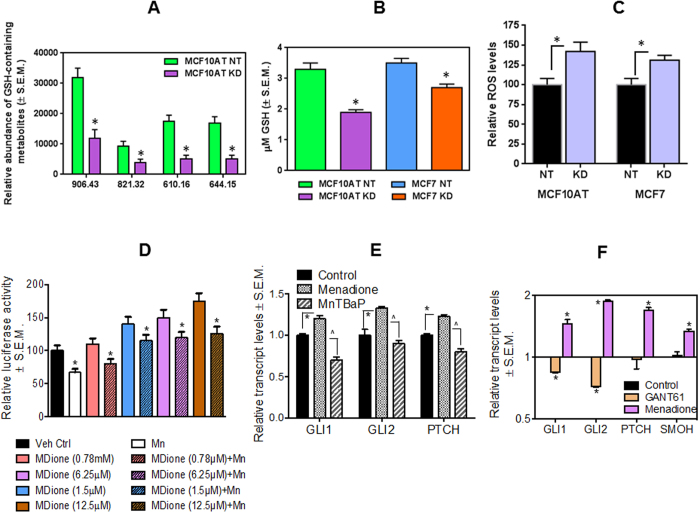
Loss of Merlin induces reactive oxygen species (ROS) concomitant with lower reduced glutathione (GSH) levels. (**A**) Four GSH containing metabolites (*m/z* 906.43, 821.32, 610.16 and 644.15) identified by global metabolomics profiling decrease in cells knocked down for Merlin. *indicates that p < 0.05. (**B**) Intracellular levels of reduced glutathione decrease in two cell lines upon the loss of Merlin, measured by enzymatic assay. *indicates that p < 0.05. (**C**) ROS levels increase in cells knocked down for Merlin (p = 0.0034 for MCF10AT KD vs. MCF10AT NT; p = 0.0019 for MCF7 KD vs. MCF7 NT). (**D**) Menadione treatment increases 8xGLI luciferase activity in MCF7 cells in a dose dependent manner. MCF7 cells were transfected with pGL3-8XGLI-Luc and treated with either Menadione or MnTBAP with different concentrations as indicated. MnTBAP was removed after 30 minutes and Menadione was added. At the end of one hour of initial treatment the medium was removed and fresh complete growth medium was added. The experiment was terminated after 8 hours. The luciferase promoter activity was normalized to total protein and represented as percentage of activity in comparison to the vehicle control. *indicates p < 0.05. (**E**) ROS induction in MCF10AT cells upregulates Hh signaling pathway. MCF10AT cells stably transfected with a non-targeting shRNA were treated with menadione to induce ROS production or MnTBAP to squelch ROS. After 24 hours of treatment, transcript expression of Hh receptor PTCH1 and transcription factors GLI1 and GLI2 was assessed by an qRT-PCR. C(t) values were normalized to GAPDH and the vehicle control (ethanol) group using ∆∆Ct method. *indicates p < 0.05. (**F**) Menadione increases, whereas GANT61 inhibits, the expression of Hh transcription factors GLI1 and GLI2 and PTCH and SMOH. The expression status of these molecules was assessed by real-time quantitative RT-PCR and normalized to endorse control gene, β-Actin. *indicates p < 0.05.

As detailed above, the relative abundance of GSH-containing metabolites is significantly decreased in cells knocked down for Merlin (KD) (Fig. 2A). In agreement with this, the intracellular levels of reduced glutathione are decreased in MCF7 and MCF10AT cells in conditions of (stable) Merlin deficiency (KD) (Fig. 2B). Recognizing the predominant role of reduced glutathione in keeping a check on cellular ROS levels (Supplementary Figure 5A), we evaluated ROS levels in cells silenced for Merlin. The steady state ROS levels show a modest but statistically significant (p < 0.05) increase in cells stably silenced for Merlin (Fig. 2C). Further increasing the cellular ROS levels with menadione resulted in significantly greater cell death of Merlin-silenced cells (Supplementary Figure 5B). This suggests that oxidative stress following Merlin deficiency is not sufficient to confound cell survival; instead an additional increase in cellular ROS renders Merlin deficient cells more susceptible to cell death.

Cellular ROS has profound effects on intracellular signaling events and plays a protective role against apoptosis^21^,^22^. Notably, oxidative stress activates Hh signaling and protects cells against apoptotic death by invoking PI-3-kinase-AKT-Bcl-2 signaling^23^,^24^. We evaluated the effects of modulating cellular ROS on the transcription activity of GLI, the transcription factor of the Hh pathway. While increasing cellular ROS with menadione had a dose-dependent increase on the transcription activity of GLI, the ROS scavenger, MnTBaP attenuated the activity of GLI in MCF7 cells (Fig. 2D). The levels of *bona fide* GLI transcriptional targets GLI1, GLI2, and PTCH also increased concordantly with an increase in ROS levels. Importantly, increases in transcriptional targets with menadione treatment were significantly decreased with the GLI transcription inhibitor GANT61 (Fig. 2E,F), indicating that signaling initiated by elevated ROS is blunted upon blocking the activity of GLI. This evidence solidified the role of ROS in facilitating activation of Hh/GLI signaling.

### Increase in cellular ROS prompted by loss of Merlin results in activated Hh signaling

**Figure 3:**
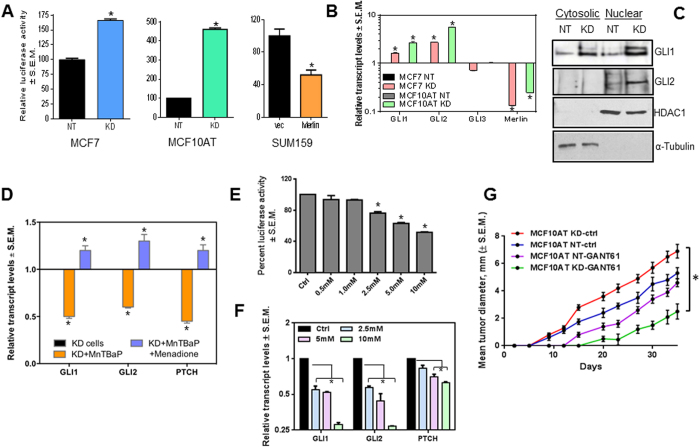
Increase in cellular ROS prompted by loss of Merlin results in activated Hh signaling. (**A**) Merlin knockdown in MCF10AT and MCF7 cells increases whereas Merlin expression in SUM159 decreases GLI luciferase activity. The promoter activity was normalized to total protein and represented as percentage of activity compared to vehicle control. *indicates statistically significant. (**B**) Silencing Merlin in MCF7 and MCF10AT cells (KD) results in a significant increase in transcript levels of GLI1 and GLI2. The expression was assessed by qRT-PCR and normalized to β-Actin. (*p < 0.05). (**C**) Merlin knockdown in MCF10AT cells increases GLI1 and GLI2 expression and facilitates nuclear accumulation of GLI1 and GLI2. Nuclear and cytosolic fractions were immunoblotted for GLI1 and GLI2. HDAC and α-tubulin were used to ascertain enrichment of the nuclear and cytosolic fractions, respectively. Shown are cropped images of the blots. (**D**) Increase in cellular ROS in Merlin-silenced cells activates Hh signaling. MCF7 cells silenced for Merlin were treated with MnTBaP or MnTBaP + menadione. Transcript levels of *bona fide* Hh transcription targets were assessed by real-time qRT-PCR. *p < 0.05 compared to vehicle control cells. (**E**) Addition of exogenous GSH counteracts the effect of Merlin deficiency on 8XGLI activity in MCF10AT KD cells (in a dose dependent manner). MCF10AT KD cells were transiently transfected with pGL3-8XGLI-Luc and treated with different concentrations of GSH. *indicates p < 0.05 compared to vehicle control cells. (**F**) GSH treatment of MCF10AT KD cells reinstates the inhibitory effect on Hh/GLI transcriptional targets. Cells were treated with different concentrations of GSH for 24 hours. Untreated MCF10AT KD cells were used as control. All experiments were done in triplicates. *p < 0.05 compared to vehicle control cells. (**G**) Merlin deficiency enforces dependence on Hh/GLI signaling. Merlin-deficient MCF10AT KD cells and non-targeting control transfected cells (MCF10AT NT) were xenografted into female athymic, nude mice and treated with either vehicle control or GANT61. Cells silenced for Merlin form tumors that grow rapidly (p < 0.05) compared to control tumors. Cells deficient for Merlin expression are effectively inhibited (p < 0.01) in their growth as tumors relative to the vehicle-treated cells. *p < 0.05 compared to vehicle control cells.

In order to attribute a direct role for Merlin in modulating Hh signaling, we assessed cells that were modulated for Merlin expression. Abrogating Merlin in MCF10AT cells (MCF10AT KD) and MCF7 cells (MCF7 KD) resulted in increased baseline activity of the Hh transcription factor GLI, measured as a luciferase readout (Fig. 3A). Conversely, SUM159 cells engineered to stably express Merlin showed a decrease in the GLI promoter activity (Fig. 3A). In concordance with the reporter activity, the steady state levels of the two important transcription factors GLI1 and GLI2 were significantly elevated in cells abrogated for Merlin (Fig. 3B). The levels of the GLI3 transcriptional repressor and SHH ligand remained unaltered. We also registered an increase in the nuclear accumulation of GLI1 and GLI2 in cells silenced stably for Merlin (Fig. 3C; Supplementary Figure 6A) clearly demonstrating that not only were the levels of GLI increased, but also the nuclear accumulation of GLI transcription factors was greater when tumor cells are modulated for Merlin deficiency.

In order to determine the impact of elevated cellular ROS on Hh/GLI activity in the KD cells, we treated cells with the ROS scavenger, MnTBaP. This caused a significant decrease in the levels of the *bona fide* GLI transcriptional targets GLI1, GLI2, and PTCH, suggesting that elevated ROS levels regulate Hh/GLI activity. Co-treatment with menadione reversed this trend (Fig. 3D). Menadione alone could not be evaluated in this assay since it compromised cell viability, similar to that depicted in Supplementary Figure 5B. We assessed the effect of supplementing cells with GSH as a means to overcome Merlin deficiency. GSH supplementation significantly reduced GLI transcriptional activity with a concordant decrease in the transcriptional targets of GLI (Fig. 3E,F). Cumulatively the data clearly indicate that loss of Merlin causes dysregulated activation of Hh/GLI signaling. Elevated ROS in cells compromised for Merlin prompts activation of Hh/GLI signaling and the anti-oxidant GSH enables reduced Hh/GLI activity in cells with Merlin deficiency.

### Targeting upregulated Hh/GLI activity is a strategy to reduce tumor burden in cells compromised for Merlin

Thus far, the data suggests that loss of Merlin causes aberrant activation of Hh signaling due to an increase in the levels and nuclear abundance of the GLI transcription factors. So we tested the impact of GANT61 on tumor growth in the perspective of Merlin deficiency in a pre-clinical system. We injected MCF10AT cells (control and silenced for Merlin) into the mammary fat pad of female athymic, nude mice. When tumors were palpable, mice were randomized and treated with GANT61 (50 mg/kg; i.p. 3 times per week). Cells silenced for Merlin formed robustly growing tumors compared to the MCF10AT NT control cells. Moreover, the tumors of MCF10AT silenced for Merlin were more responsive to GANT61 showing a notably decreased tumor growth rate (Fig. 3G).

We enriched the tumor cells from the mice treated with GANT61 and evaluated the effect of GANT61 on their viability in culture. Tumors from the MCF10AT KD cells showed a stark sensitivity to an increasing dose of GANT61 relative to the MCF10AT NT cells, which were resistant to GANT61 (Supplementary Figure 6B). This *ex vivo* data complements the *in vivo* findings and strongly attests targeting Hh/GLI signaling in cells that have lost Merlin expression. Thus, loss of Merlin enforces dependence on active Hh signaling for *in vivo* tumor growth.

### Loss of Merlin manifests as enhanced mesenchymal phenotype that is sensitive to inhibition by GANT61

**Figure 4:**
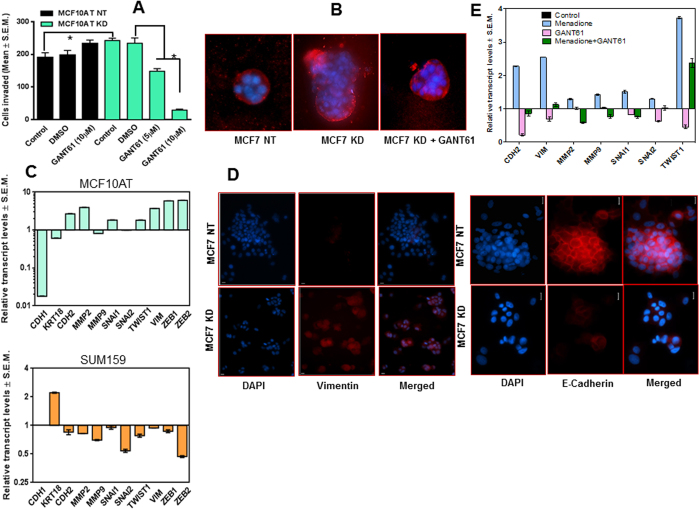
Loss of Merlin prompts mesenchymal attributes through modulation of Hh signaling. (**A**) Merlin-deficient MCF10AT cells (KD) demonstrate significantly decreased (p < 0.01) invasion when treated with GANT61. (**B**) Loss of Merlin corroborates with loss of integrity of a basement membrane as evidenced by laminin-V staining that was rescued by GANT61 indicating that blocking Hh signaling attenuates invasive phenotype manifested upon loss of Merlin. Acinar structures formed by Merlin-silenced cells in a three-dimensional matrix were stained with anti-laminin-V antibody (red) to visualize the basement membrane. Nuclei are stained with DAPI (blue). (**C**) Merlin expression in SUM159 cells blunted mesenchymal attributes (N-Cadherin [CDH2], MMP-2, MMP-9, SNAI1, TWIST1, Vimentin, ZEB1 and ZEB2); abrogation of Merlin blunted the epithelial attributes (E-cadherin [CDH1] and Keratin 18) concomitant with a gain in the mesenchymal phenotype in MCF10AT cells. The graphs represent data analyzed to compare SUM159 (vector control) cells *versus* the SUM159 cells engineered to express Merlin and the MCF10AT (non-targeting) control cells *versus* the MCF10AT cells silenced for Merlin. Data was acquired by quantitative RT-PCR data and normalized to β-actin expression relative to vector control. (**D**) Immunocytochemistry of MCF7 cells knocked down for Merlin shows an increase in Vimentin expression with a concomitant decrease in E-Cadherin expression. Nuclei are stained with DAPI (blue). The bar indicates 10 μm. (**E**) The GLI inhibitor GANT61 antagonized the effects of menadione on increase in mesenchymal attributes. MCF7 cells treated with Menadione showed an increase in EMT markers that were thwarted by the GLI transcription inhibitor GANT61.

Hh signaling and oxidative stress stimulate invasiveness of tumor cells^25^,^26^. Cells knocked-down for Merlin showed a significant increase (p < 0.05) in their invasive potential (Fig. 4A, Supplementary Figure 6C). However, treatment with the small molecule GANT61, an inhibitor of GLI transcription activity, substantially decreased invasion (p < 0.01) in a dose-dependent manner (Fig. 4A). We also assessed the behavior of these cells in 3D culture. In contrast to the MCF7 NT and MCF10AT NT cells that form a distinctly circumscribed structure surrounded by intact basement as visualized with laminin V, the integrity of the laminin enclosure was breached in the cells silenced for Merlin. Importantly, treatment of the MCF7 KD and MCF10AT KD cells with GANT61 inhibited their invasive phenotype and restored the integrity of the laminin V-enclosed structure (Fig. 4B, Supplementary Figure 7A). This indicated that the invasive manifestation evoked by the loss of Merlin can be blunted by treatment with a Hh/GLI inhibitor.

Given the vital role of Hh activity in epithelial-mesenchymal plasticity that we and others have reported^27^,^28^,^29^,^30^, we queried the expression of proteins indicative of the epithelial or mesenchymal characteristics. Merlin deficiency in MCF10AT and MCF7 cells was associated with a gain in the expression of mesenchymal markers concomitant with a decrease in expression of epithelial markers (Fig. 4C; Supplementary Figure 7B). In contrast, expression of Merlin in SUM159 cells was associated with blunted expression of mesenchymal markers (Fig. 4C). This was also strikingly visible in the MCF7 cells silenced for Merlin where the loss of Merlin resulted in increased expression of vimentin protein concomitant with loss of E-cadherin (Fig. 4D).

In the perspective of our findings that an increase in cellular ROS activates Hh signaling, we evaluated a role for cellular ROS in modulating epithelial-mesenchymal-plasticity. An increase in cellular ROS following menadione treatment caused a predominant increase in multiple mesenchymal markers. The GLI inhibitor, GANT61 blunted the effects of menadione on the expression of mesenchymal markers attesting that the mesenchymal attributes elicited by menadione-induced ROS were attributable, in part, to Hh/GLI activity (Fig. 4E). Collectively, the findings clearly indicate that the effects of loss of Merlin on manifestation of mesenchymal attributes are phenocopied by the increase in cellular ROS; inhibiting the activity of GLI attenuates the mesenchymal attributes. Collectively, the data suggest that loss of Merlin enables a mesenchymal phenotype that is sensitive to inhibition by GANT61.

### GLI1 enhances tissue identification and discriminatory power of Merlin

**Figure 5:**
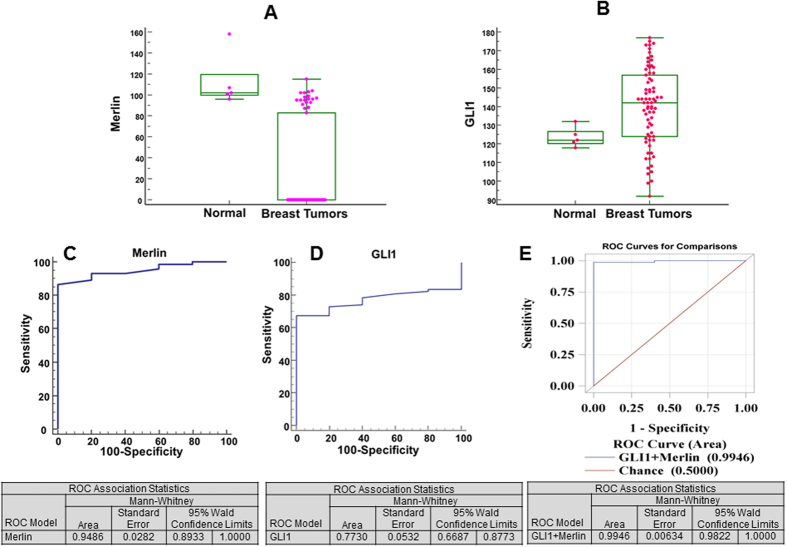
GLI1 enhances tissue identification and discriminatory power of Merlin. (**A** and **B**) Breast tumor tissues showed significantly decreased staining intensity for Merlin (**A**) and significantly elevated staining intensity for GLI1 (**B**). (**C** and **D**) Logistic regression model applied to a binary variable of normal and tumor tissue to the data demonstrates the discriminatory power of Merlin (**C**) and GLI1 (**D**). (**E**) GLI1 mitigates the discriminatory power of Merlin.

We evaluated primary breast tumors from patients for their expression of Merlin and GLI1 by immunohistochemistry. In contrast to Merlin expression in normal breast tissues, tumor specimens showed significantly decreased staining for Merlin (p < 0.0001) (Fig. 5A; Supplementary Figure 7C). GLI1 expression was notably increased (p < 0.043) in tumor tissues relative to normal tissues (Fig. 5B; Supplementary Figure 7C). These relationships were independent of hormonal status, age, and tumor grade. To assess the discriminatory power of Merlin and GLI1, we applied a logistic regression model to a binary variable of normal and tumor tissue to our data. The Chi-square test for the ROC curve not being equal to chance alone (*p* < *0.001*; ROC curve area = 0.9486; 95% CI for the ROC curve (0.8933, 1.0)) indicates that Merlin has a discriminatory power for distinguishing between normal and tumor tissues (Fig. 5C). The logistic regression also showed that GLI1 by itself has appreciable discriminatory power between normal and tumor tissues (*p* < *0.001*; ROC curve area = 0.7730; 95% CI for the ROC curve (0.6687, 0.8773)) (Fig. 5D). Further, multiple logistic regression showed that GLI1 increases the discriminatory power of Merlin (*p* < *0.001*; ROC curve area = 0.9946; 95% CI for the ROC curve (0.9822, 1.0) (Fig. 5E). Cumulatively, these data clearly present a compelling case for Merlin deficiency and gain of GLI1 expression.

## Discussion

### Loss of Merlin decreased levels of reduced glutathione in tumor cells

As a tumor suppressor, Merlin has been characterized for its role in mediating contact inhibition of cell growth while eliciting the involvement of receptor tyrosine kinase, PI3K/Akt, small GTPases, cell adhesion, mammalian target of rapamycin (mTOR), and Hippo pathways. Given the convergent role of Merlin in inhibiting cell proliferation, we undertook an unbiased, untargeted metabolomics approach to discern the global metabolomic profile impacted by Merlin in breast cancer cells. Of the more than 6500 metabolites detected via our untargeted LC-MS metabolomics approach, appropriate technical filters allowed for the discernment of 47 metabolites that were significantly changed upon modulation of Merlin expression in breast cancer. None of these metabolites matched the available MS/MS spectra with reference to publically accessible human metabolite databases, underscoring the novelty of these metabolites. We minimized this limitation by utilizing the mummichog functional grouping approach to pathway analysis^31^. Mummichog pathway analysis revealed that alterations in Merlin levels broadly affected metabolism of glutathione, amino acids, nucleotides, and vitamins. By analyzing MS/MS spectra, we were able to confirm that Merlin alters the levels of various metabolites containing amino acid derivatives and nucleotide derivatives.

In the present study, while the precise identification of metabolites was challenging due to their low abundance and unavailability of references, we identified that Merlin-modulates glutathione levels in breast cancer. The levels of reduced glutathione were significantly decreased in cells knocked down for Merlin as evident by the decreased abundance of the corresponding GSH-containing metabolites.

### Increase in cellular ROS levels following Merlin deficiency resulted in increased activity of Hh/GLI signaling

Glutathione plays a significant role in cellular progression, cell differentiation, and apoptosis. Relevant to this study is the fact that GSH plays a main role in the maintenance of the intracellular redox balance, mainly by antioxidant defense against ROS and against lipid hydroperoxidases^32^. At low levels, ROS act as signaling molecules and activate pathways favoring cell survival and proliferation, while at moderate levels ROS induces DNA damage and promotes mutagenesis. Extreme levels of ROS levels exert oxidative stress on the cells that can lead to cell death or senescence^33^. The antioxidant function of GSH is determined by the redox-active thiol (−SH) of cysteine that is oxidized when GSH reduces its target proteins/molecules. In the process GSH is oxidized to GSSG. As such, the GSH:GSSG ratios are important to maintain the redox balance in cells. The modulation of GSH is viewed paradoxically. While enhancing the capacity of GSH protects cells from redox-related changes, the strategy of depleting GSH is aimed at sensitizing cancer cells to chemotherapy. The approach of inhibiting GSH in combination with standard-of-care chemotherapies is underway in clinical trials^34^,^35^. As such, our data indicate that by virtue of decreasing levels of reduced GSH, loss of Merlin enables an increase in the cellular levels of ROS. From the perspective that Merlin expression is severely reduced in advanced breast cancer and that increased ROS is associated with metastatic potential, our data uncover a novel role for Merlin in regulating tumor cell redox balance and ROS levels.

ROS modulates multiple intracellular signaling events including PI3K/AKT, NF-κB, STATs, Src, Wnt/β-catenin and others^21^,^36^. Our data clearly demonstrate that ROS causally increases the activity of Hh/GLI signaling characterized by upregulation of the transcriptional activity of the GLI transcription factors. In cells abrogated for Merlin, increased levels and nuclear accumulation of the Hh transcription factors GLI1 and GLI2, corroborated with greater GLI activity and manifestation of a mesenchymal phenotype characterized by loss of a distinct basement membrane structure. While Hh/GLI signaling is classically activated by specific Hh ligands, non-canonical activation of GLI (that bypasses the need for specific Hh ligands) is well-reported to occur through the activities of EGF, OPN, TGF-beta^37^,^38^,^39^. Activation of Hh signaling as a protective effort by neuronal cells in response to oxidative stress engages the cellular anti-apoptotic machinery^23^,^24^. Our data shed critical insight into the molecular mechanistic events in this process. Increase in cellular oxidative stress following loss of Merlin resulted in increased levels of the GLI1 and GLI2 transcription factors and their ensuing transcriptional activity.

### Merlin deficiency sensitizes breast cancer cells to inhibition of Hh/GLI signaling

Remarkably, cells abrogated for Merlin showed remarkable sensitivity to the GLI inhibitor GANT61. While the growth of cells silenced for Merlin was overall greater than control cells, cells abrogated for Merlin also showed the greatest response to GANT61, presenting evidence that cells compromised for Merlin acquire dependence on Hh/GLI signaling. GANT61 also resulted in reversal of the invasive and mesenchymal phenotype. This effect was phenocopied in cells induced for cellular ROS with menadione, where GANT61 antagonized the effects of menadione-mediated ROS increase on the mesenchymal phenotype. Collectively, these observations underscored the fact that increased ROS following loss of Merlin sensitizes cells to inhibition of Hh/GLI signaling. In our cohort of breast tumor tissues, GLI1 enhanced tissue identification and discriminatory power of Merlin, cumulatively presenting as powerful substantiation of the relationship between these two proteins.

## Conclusion

In conclusion, the approach of unbiased, untargeted metabolomics revealed a novel function of Merlin on glutathione regulation. The resultant increase in cellular ROS profoundly impacted activation of Hh/GLI signaling, likely as a survival mechanism. This is evident in the finding that increased GLI1 expression enhances the discriminatory power of Merlin in human breast tissues. The activation of Hh signaling in Merlin-deficient conditions also presents as the tumor cells’ vulnerability since inhibiting Hh/GLI signaling in Merlin-deficient breast tumor cells limits their oncogenic growth. Notably, our evidence indicates that targeting Hh/GLI signaling may be a viable therapeutic strategy in breast tumors that are deficient in Merlin expression.

## Materials and Methods

### Cell Culture

SUM159 and MCF10AT cells were cultured as previously described^1^,^40^. MCF7 cells were cultured in DMEM/F-12 (1:1) (Life Technologies, Carlsbad, CA) supplemented with 10% heat-inactivated FBS (Atlanta Biologicals, Lawrenceville, GA) with 5 μg/mL insulin without antibiotics or antimycotics in a humidified 5% CO_2_ environment. The generation of stable Merlin-expressing transfectants of SUM159 is previously described^1^. Four shRNA sequences targeting *NF2* (gene encoding Merlin) and a scrambled control shRNA were purchased from Oligoengine (Seattle, WA) and cloned into pSuperior.GFP.neo vector. The non-targeting scrambled control (NT) or each shNF2 plasmid was transfected into MCF7 cells and cells stably knocked down for Merlin (KD) were selected. Similarly, MCF10AT cells stably knocked-down for Merlin were generated by transfecting pLenti-H1-si631-NF2-U6-CMV-GFP-2A-Puro or scrambled control (Capital Biosciences, Rockville, MD). The *NF2*-targeting si/shRNA sequences used are as follows:

5′ AGAUACUGACAUGAAGCGG 3′; 5′ AUACUGACAUGAAGCGGCU 3′;

5′ GGCAGCAGCAAGCACAAUA 3′; 5′ GCAGCAAGCACAAUACCAU 3′.

### LC-MS/MS analysis

An aliquot (5 μL) of each sample was loaded onto a Nano cHiPLC 200 μm ID × 0.5 mm ChromXP C18-CL 3 μm 120 Å reverse-phase trap cartridge (Eksigent, Dublin,CA) at 2 μL/min using an Eksigent autosampler. The bound peptides were flushed onto a Nano cHiPLC column 200 μm ID ×15 cm ChromXP C18-CL 3 μm 120 Å column (Eksigent) with a 15 min linear (5–95%) acetonitrile gradient in 0.1% formic acid at 1000 nL/min using an Eksigent 415 NanoLC system. The column was washed with 95% acetonitrile-0.1% formic acid for 5 min and then re-equilibrated with 5% acetonitrile-0.1% formic acid for 5 min. A 5600 Triple-Tof mass spectrometer (Sciex, Toronto, Canada) was used to analyze the metabolite profile. The IonSpray voltage for positive and negative modes were +/−2300 V and the declustering potential was +/−80 V. Ionspray and curtain gases were set at 10 psi and 25 psi, respectively. The interface heater temperature was 120 °C. Eluted compounds were subjected to a time-of-flight MS survey scan from *m/z* 50–1000 to determine the top twenty most intense ions for MSMS analysis. Product ion time-of-flight scans at 50 msec using a collision energy spread of 15 eV with a set collision point of 35 eV were carried out to obtain the tandem mass spectra of the selected parent ions over the range from *m/z* 50–1000. Spectra were centroided and de-isotoped by Analyst software, version 1.6 TF (Sciex).

### Data Analysis and Metabolite Identification

LC-MS data were processed using XCMSonline (https://xcmsonline.scripps.edu; Scripps, La Jolla). Each sample was normalized to total ion chromatogram. Peaks were manually filtered to retain median retention time between five and 25 min and mass tolerance was set to 0.01 Da. Excel.csv files were created containing the median mass and retention time for each peak and its peak area. These were uploaded for statistical analysis using MetaboAnalyst 3.0 (http://www.metaboanalyst.ca). Data were normalized by the total ion current of the filtered metabolite ions, mean centered and subjected to Pareto scaling. Using univariate analysis, a fold change less than 0.5 or greater than 2, and p-value < 0.05 (Student’s t-test) was considered significant for further analysis. All peaks passing the initial filters were classified according to “real” or “not real” peaks based on visual confirmation of their ion chromatograms. Mass to charge ratios were searched via the METLIN database (Scripps) assuming an accuracy of 10 ppm for matches to previously identified metabolites with available MS/MS spectra. Similar searches were conducted on the Human Metabolite Database (HMDB)^41^,^42^,^43^ Pathway analyses were conducted via the raw, unfiltered data using “Connections” feature of XCMSonline which utilizes the Mummichog metabolite functional grouping approach^31^. Mummichog analysis was also conducted at the command line level on the normalized data from the MetaboAnalyst procedure using Mummichog, version 1.0.5 (https://code.google.com/p/atcg/wiki/mummichog_for_metabolomics). The data files (in Excel.txt format) contained the *m/z* of all the metabolite ions, their retention times, p-values, and t-scores.

To identify metabolites that were significantly changed upon modulation of Merlin, three data analysis approaches were employed. First the parent or precursor ions and retention times (Rt) of all 47 metabolites altered in the context of Merlin were queried for potential matches in METLIN and the Human Metabolome Database. None of these metabolites could be identified by this method. Because the identity of these metabolites was not known, conventional pathway analysis was not possible. The mummichog analysis within XCMSOnline revealed likely changes in the metabolism of glutathione and a broad range of amino acids (Supplementary Table 2). As no structural information was obtained after exhaustive database search, accurate masses of selected precursor and product ions together with detailed interpretation of fragmentation patterns of precursor ions upon MS/MS enabled us to propose partial identification of the altered metabolites.

### Western Blotting Analysis

Cell lysates were collected in NP-40 buffer. Cytosolic and nuclear fractions were collected with an NE-PER kit (Pierce, Rockford, IL). Antibodies used include GLI1 (#2643; Cell Signaling, Danvers, MA), Merlin (#sc-55574; Santa Cruz Biotechnology, Santa Cruz, CA), GLI2 (#PA1941; Boster Bio Pleasanton, CA), GAPDH (#2118; Cell Signaling). Blots were developed with SuperSignal substrate (Pierce). The cytosolic and nuclear fractions were confirmed with anti-β-tubulin (#2146; Cell Signaling) or anti- HDAC1 (#2062; Cell Signaling) respectively. To assess the expression and localization of GLI1 and GLI2 in the MCF10AT KD cells (knockdown for NF2) in comparison to the MCF10AT NT (transfected with non-targeting vector), 100 μg of cytosolic and nuclear lysates were immunoprecipitated with either GLI1 or GLI2 antibodies and immunoblotted with the corresponding antibodies.

### Luciferase Assay

Cells (30,000) were transfected with an 8XGLI construct in pGL3 promoter plasmid as described previously (3). Empty pGL3 promoter vector was used as control. Readings were normalized to total protein content. Each parameter was studied in triplicate and the experiment repeated at least three times. To evaluate the effect of reduced glutathione (ROS scavenger) on GLI activity, MCF10AT KD cells were transiently transfected with the 8XGLI construct and the medium was supplemented with different concentrations of GSH (L-Glutathione reduced, *Cat. No.* 5219 TOCRIS). The experiment was terminated after 24 hours of GSH supplementation.

### Quantitative RT-PCR (qRT-PCR)

qRT-PCR was done in triplicate and repeated at least once. TaqMan Gene Expression Assays (Applied Biosystems) were used to query the transcripts of interest. Transcript levels were normalized to endorse control gene GAPDH or β-actin (ΔCT) to calculate changes in expression (2^ΔΔ^CT).

### MTS Assay

Cells were seeded in 96-well plates at a density of 10,000 cells/well. The effect of menadione (and ethanol as vehicle control) on cell viability was assessed by MTS assay (Promega, Madison, WI) after 24 hours of treatment.

### Glutathione assay

Cells were seeded in 96-well plates overnight; 100 μL of the GSH-Glo reagent (Promega, Madison, WI) was added to each well, mixed, and the plate was incubated at room temperature for 30 minutes. Luciferin Detection Reagent was added to each well and luminescence was recorded.

### ROS assay

Cells were treated as follows: Control, 100 μM MnTBAP (30 mins), 100 μM MnTBAP (30 mins) +100 μM Menadione (1 hour), or a 100 μM Menadione only (1 hour) group. CellRox Deep Red Reagent (LifeTechnologies) was used to detect oxidative stress and analyzed using a BD LSRII Analyzer.

### Immunocytochemistry

Plated cells were fixed in 4% paraformaldehyde, permeabilized with 0.3% Triton X-100 followed by labeling with either anti-E-Cadherin (#3195, Cell Signaling) or anti-Vimentin (#5741, Cell Signaling) or anti-GLI1 primary antibody (#2643, Cell Signaling) overnight at 4 °C. Images were captured at 40X, and the acquisition time (1 second for E-Cadherin and 4 seconds for Vimentin and GLI1) was kept constant across compared groups.

### 3D Cell Culture

Cells were seeded in 400 μl of growth media supplemented with 2% 3D Culture Matrix (Cultrex 3-D Culture Matrix Reduced Growth Factor Basement Membrane Extract, Trevigen, Gaithersburg, MD) in chamber slides. Laminin-V was visualized using anti-laminin-V antibody (#MAB19562, Millipore) followed by secondary antibody (#A21125, Alexa Fluor 594 goat anti-mouse IgG; 1:1000, Molecular Probes) and nuclei were stained with Vectashield Hard Set Mounting Medium with DAPI (Sigma, St. Louis, MO).

### Invasion assay

Cells were treated for 24 hours with DMSO, GANT61 or were left untreated. BD Biocoat Matrigel invasion chambers were thawed and rehydrated prior to seeding 20,000 cells/chamber in serum-free medium containing DMSO or GANT61 concordant with cell treatment. The assay was performed in triplicate and repeated twice as previously described^44^.

### Animal Studies

The animal studies were approved by the IACUC at UAB. All methods were performed in accordance with relevant guidelines and regulations. Cells (5 × 10^6^) were injected into the inguinal mammary fat pad of 6-week old female, athymic nude mice 3–5 days following implantation of an estrogen pellet (#SE-121:0.72 mg/pellet dose for 60 day release time; Innovative Research of America) in the sub-scapular region. GANT61 administration was initiated 8 days after cell injection and tumor measurements were recorded three times per week. The experiment was terminated at day 35. Tumor cells were harvested from xenografts and assessed for sensitivity to GANT61 by MTS assay.

### Immunohistochemistry

Breast tumor tissue microarrays were procured from NCI Cooperative Breast Cancer Tissue Resource and stained immunohistochemically for Merlin (#A-19; Santa Cruz) and GLI1 (#ab7523; Abcam). Staining intensity was quantitated with computer-assisted image analysis in a Dako ACIS III Image Analysis System (Glostrup, Denmark)^45^.

### Statistical analyses of *in vitro, in vivo* and patient data

Statistical differences between groups were assessed using the Mann-Whitney test, t-test or ANOVA, using GraphPad Prism 4 software or SAS software version 9.4 (SAS Inc.). All results with a *p* value less than 0.05 were considered statistically significant.

Associations between intensities of Merlin and GLI expressions and patient Breast Cancer status data were assessed using the Wilcoxon rank test. The univariate and multiple logistic regression models were fit to a binary variable normal *versus* tumor with Merlin and GLI1 as possible predictors. The possibility of developing a model using the relationship between GLI1 and Merlin was tested with a logistic regression model. The area under the receiver operating characteristic (ROC) curve and the corresponding 95% confidence interval was used to determine the predictive ability of models and in model selection. The Chi-square test was used to test the ROC curve not being equal to chance alone.

## Additional Information

**How to cite this article**: Das, S. *et al*. Loss of Merlin induces metabolomic adaptation that engages dependence on Hedgehog signaling. *Sci. Rep.* **7**, 40773; doi: 10.1038/srep40773 (2017).

**Publisher's note:** Springer Nature remains neutral with regard to jurisdictional claims in published maps and institutional affiliations.

## Supplementary Material

Supplementary Information
