# Supplementary material for: Loss of Merlin induces metabolomic adaptation that engages dependence on Hedgehog signaling
Source: Sci Rep. 2017 Jan 23;7:40773. doi: 10.1038/srep40773 (PMC5256100; doi:10.1038/srep40773)

Loss of Merlin induces metabolomic adaptation that engages dependence on Hedgehog signaling

Running title: Merlin modulates metabolomic adaptation

Shamik Das[a*], William P. Jackson[a*], Jeevan K. Prasain[b*], Ann Hanna[a], Sarah K. Bailey[a], J. Allan Tucker[f], Sejong Bae[c,d], Landon S. Wilson[e], Rajeev S. Samant[a,c], Stephen Barnes[b,c,e], Lalita A. Shevde[a,cƗ]

[a]Department of Pathology, [b]Department of Pharmacology and Toxicology, [c]Comprehensive Cancer Center, [d]Department of Medicine, [e]Targeted Metabolomics and Proteomics Laboratory, University of Alabama at Birmingham, Birmingham, AL 35233; [f]University of South Alabama.

*Contributed equally to this work

Ɨ Corresponding author: Lalita A. Shevde, 1824 6th Ave S, WTI320D, University of Alabama at Birmingham, Birmingham, AL, 35233; Tel: (205)-975-6261; Email: lsamant@uab.edu

SUPPLEMENTARY RESULTS

Endogenous phosphorylated metabolites: We identified three phosphorylated metabolites significantly changed (>2 fold) with modulation of Merlin. The deprotonated molecular ion *m/z* 453.0657 (Rt 7.14 min) gave rise to the product ion m/z 355.0861 due to the loss of 98 Da (H_3PO_4) (**Supplementary Figure 4A**). The presence of phosphate moiety is further supported by the product ions *m/z* 78.9589 (PO_3^- calcd.78.9591) and 96.9693 ($H_2PO_3^-$ calcd. 96.9693). Neutral losses of H_2O (18 Da) and 132 Da from the precursor ion *m/z* 453.0657 yielded product ions of *m/z* 435.0601 and 320.0325, respectively. The characteristic neutral loss of 132 Da is an indicative of a ribose sugar. The presence of product ions due to the loss of glycosidic moiety and the phosphoester bond cleavage indicated that this metabolite is likely a phosphorylated nucleotide. The other two phosphorylated metabolites with protonated molecular ions at *m/z* 316.139 and 492.1378 showed characteristic phosphate moiety losses. The MS/MS fragmentation of the precursor ion *m/z* 316.139 $[M+H]^+$ generated product ions due to losses of 80 Da (HPO_3) and 98 Da (H_3PO_4) giving rise to 236.1257 and 218.1149, respectively (**Supplementary Figure 4B**). Similarly, the precursor ion *m/z* 492.1378 $[M+H]^+$ showed product ions *m/z* 474.1250 and 412.1266 due to the losses of H_2O (18 Da) and HPO_3 (80 Da) (**Supplementary Figure 4C**), respectively, indicating this metabolite to be a phosphorylated metabolite.

We also detected a metabolite conjugated with O-linked N-acetylglucosamine (*O*-GlcNAc) with a doubly charged *m/z* 386.7123 in positive ion mode. Fragmentation of this ion *m/z* 386.7123 displayed the characteristic product ions corresponding to HexNAc oxonium ions *m/z* 204.0869 $[C_8H_{14}O_5N]^+$, and other five signature ions *m/z* 186.0760 $(C_8H_{12}O_4N]^+$, 168.0665 $[C_8H_{10}O_3N]^+$, 144.0652 $[[C_6H_{10}O_3N]^+$, 138.0539 $[C_7H_8O_2N]^+$, and 126.0547 $[C_6H_8O_2N]^+$ (**Supplementary Figure 4D**) (25). Even though complete identification of the *O*-GlcNAc site onto amino acid residues of a peptide was not possible in the present studies, the immonium ion of serine was

observed at *m/z* 60.0448. (calcd. 60.0444). The product ion *m/z* 84.036 is likely derived from glutamine residue.

SUPPLEMENTARY INFORMATION-COMPLETE LEGEND FOR FIGURE 1

Complete Legend for Figure 1: Negative ion product ions spectra of GSH containing metabolites. The proposed GSH conjugates were structurally divided into three classes:

Thioester GSH conjugates: Three thioester class GSH metabolites with *m/z* 821.3264, 906.4371 and 468.1325 were observed.

(A): The ESI-MS/MS spectrum of *m/z* 821.3264 precursor ion. The product ion spectrum of this ion showed a neutral loss of 307 Da and prominent product ion fragments at *m/z* 306.0739 as *m/z* 272.0877, 254.0739, 210.0907, 179.0504, 160.0111, 143.0447 and 128.0363. This fragmentation pattern is very similar to that of the glutathionyl-derived species observed in the negative ion mode [46]. The presence of product ions *m/z* 306.0739 and 272.0877 together indicated this metabolite likely to be a thioester GSH conjugate. The structure of carbonyl carbon moiety in which GSH is conjugated is unknown. The product ion *m/z* 514.2514 generated after the neutral loss of 307 Da from the precursor ion *m/z* 821.3264 corresponds to a reactive endogenous cancer metabolite that forms a stable conjugate with GSH. The other GSH conjugates appeared at *m/z* 906.4371 and 468.1325 and their product ion spectra are dominated by the same ions characteristic of thioester GSH conjugates.

(B) The MS/M:S spectrum of *m/z* 906.4371. The neutral loss of 307 Da from *m/z* 906.4371 gave rise to the product ion *m/z* 599.3226. These pieces of information indicated that this metabolite has two major substructures corresponding to GSH (*m/z* 306.0739) and *m/z* 599.3226 (parent metabolite). The precursor ion *m/z* 468.1325 also showed characteristic product ions corresponding to GSH in its product ion spectrum (data not shown). The presence of a product ion *m/z* 306.0761 after a neutral loss of 162.0 Da indicated the GSH moiety is likely to be conjugated with a hexose sugar.

Disulfide GSH conjugates: We observed two disulfide GSH conjugates (*m/z* 610.1654 and 644.1570) of unknown structures.

(C): The product ion spectrum of *m/z* 610.1654. A series of product ions corresponding to glutathionyl-derived species were observed. In addition, two characteristic ions *m/z* 338.0389 and 304.0613 were observed in the product ion spectrum. The ion *m/z* 338.0389 can be formed by cleavage of the C-S bond (GSS^-), while the ion *m/z* 304.0613 corresponds to a dehydrogenated GS- ion [46]. Unlike oxidized GSH (GSSG), the precursor ion *m/z* 610.1654; is one mass unit less than that of GSSG. This revealed a possibility of modification in one of the amino acids in the GSH moiety.

Similarly, MS/MS of *m/z* 644.1570 generated intense product ions with *m/z* 339.1021, 306.0750, 338.0378, 306.0750 and 304.0614. The ions *m/z* 306.0750 and 338.0378 represent deprotonated tripeptide persulfides. Based on these mass spectrometric data, both metabolites (*m/z* 610.1654 and 644.1570) were identified as disulfide GSH conjugates.

(D): MS/MS spectra *m/z* 571.2096.

Benzylic GSH conjugate: The $[M-H]^-$ precursor ion *m/z* 571.2096, upon MS/MS fragmentation, produced ions *m/z* 128.0366, 143.0463, 179.0491, 210.0900, 254.0796 and 272.0900, representing the glutathionyl moiety. The absence of product ion m/z 306 in the spectrum indicated that the product ions are generated exclusively from the cleavage of bonds within the gluthathionyl moiety [47]. The loss of 129 Da can be rationalized by the elimination of a neutral pyroglutamic acid moiety giving rise to *m/z* 442.1760. These pieces of information indicate that this metabolite is likely a benzylic GSH conjugate.

SUPPLEMENTARY DATA - Legends

Figure S1: (A) Schematic of metabolite extraction and sample preparation for nano LC-MS. **(B)** Immunoblot demonstrating MCF10AT and MCF7 cells engineered to knock down Merlin and SUM159 cells stably expressing Merlin (NT: non-targeting control plasmid; KD: stable knock down of Merlin; vec: empty vector control plasmid).

Figure S2: Modulation of Merlin protein levels alters the breast cancer metabolome.

(A-D): Ions detected by nanoLC-MS were normalized to total ion concentration and subjected to a series of *in silico* filters (see Methods) to select for metabolites altered in the context of Merlin re-expression **(A&B)** or loss of Merlin **(C&D).**

Circle radius represents fold change from control cells and color intensity represents p-value. Green metabolites are downregulated, while red metabolites are upregulated in altered cells.

Figure S3: The total ion chromatogram is similar between cells modulated for Merlin expression. Shown are **(A)** SUM159-vector control cells, **(B)** SUM159 cells expressing Merlin, **(C)** MCF10AT scrambled control cells, **(D)** MCF10AT cells knocked down for Merlin.

Figure S4: MS/MS spectra of three phosphorylated and one O-GlcNAc-ylated metabolite altered in the context of Merlin. Negative ion product ions MS/MS spectra of *m/z* 453.0657 (Panel **A**). Positive MS/MS spectrum of *m/z* 316.1390 (Panel **B**). Positive product ions obtained from MS/MS spectra of *m/z* 492.1348 (Panel **C**). Positive MS/MS spectrum of *m/z* 569.3487 (Panel **D**).

Figure S5: (A): Schematic of the effect of the loss of Merlin on breast cancer cell GSH levels. Lower GSH levels may be responsible for the observed accumulation of ROS (H_2O_2 is cited as an example of ROS inducer). The level of ROS in MCF10AT and MCF7 cells was quantified in a

fluorescence plate reader. **(B):** MCF7 and MCF10AT cells (control and abrogated for Merlin) were evaluated for their response to menadione by an MTS assay. Abrogation of Merlin renders the cells more sensitive to menadione. *indicated $p<0.05$.

Figure S6: (A): Merlin knockdown in MCF7 cells increases GLI1 expression and enables greater nuclear presence when evaluated by immunocytochemistry. **(B):** MCF10AT control and Merlin-silenced (KD) cells were recovered *ex vivo* from the GANT61-xenograft tumors and assayed for their sensitivity to GANT61. Cells abrogated for Merlin display greater sensitivity to GANT61. Cell viability was assessed by an MTS assay. **(C):** The MCF7 cells silenced for Merlin show increased invasion through Matrigel. *indicates statistically significant differences relative to respective control ($p<0.05$).

Figure S7: (A): Merlin-silenced MCF10AT (KD) cells were grown in a three-dimensional matrix comprised of basement membrane extracts. Acinar structures were fixed in paraformaldehyde and stained with anti-laminin-V antibody (red) to visualize the basement membrane. Nuclei are stained with DAPI (blue). MCF10AT NT represents the control group and MCF10AT KD+GANT61 represent MCF10AT KD cells treated with GANT61. Arrows indicate invasive growth into the matrix and places where basement membrane is compromised. **(B):** Merlin silencing in MCF7 cells decreased epithelial marker E-cadherin and upregulated expression of mesenchymal transcription markers MMP-2, MMP-9, SNAI1, SNAI2, TWIST1 and Vimentin. Quantitative RT-PCR data are normalized to β-actin expression and fold changes in expression (log10) are relative to vector control. **(C):** Representative immunohistochemical staining of normal breast tissue and breast tumor tissue for GLI1 and Merlin.

Supplementary Table 1: Summary of untargeted LC-MS experiments in cells re-expressing Merlin (SUM159 Merlin) and cells knocked down for Merlin (MCF10AT shMerlin) after filtering. 47 metabolites were identified as significantly altered in the context of Merlin in breast cancer.

Supplementary Table 2: Mummichog pathway analysis suggests significant overlap between metabolites altered in the context of Merlin and known metabolite pathways. **(A & B):** Pathways with significant overlap in SUM159 cells re-expressing **(A)** and MCF10AT knocked down for **(B)** Merlin. Pathways significant and overlapping in both systems are highlighted with color.

Supplementary Table 3: Re-expression of Merlin in SUM159 cells alters seven metabolites. **(A &B):** Ions showing a fold change of greater than two from ESI positive mode **(A)** and negative mode **(B)**.

Supplementary Table 4: Abrogation of Merlin in MCF10AT cells alters the levels of 40 metabolites. **(A &B):** Ions showing a fold change of greater than two from **(A)** ESI positive mode and **(B)** negative mode.

Supplementary Table 5: Metabolites detected in ESI positive mode in the context of Merlin. Three metabolites were significantly altered in the context of Merlin re-expression and 14 metabolites in the context of knock down of Merlin. There was no overlap between the metabolites changing in the perspective of loss of Merlin in the MCF10AT cells or the re-expression of Merlin in SUM159 cells.

Supplementary Table 6: Metabolites detected in ESI negative mode in the context of Merlin. Four metabolites were significantly altered in the context of Merlin re-expression and 26 metabolites in the context of knock down of Merlin. There was no overlap between the

metabolites changing in the perspective of loss of Merlin in the MCF10AT cells or the re-expression of Merlin in SUM159 cells.

Supplementary Figure 1

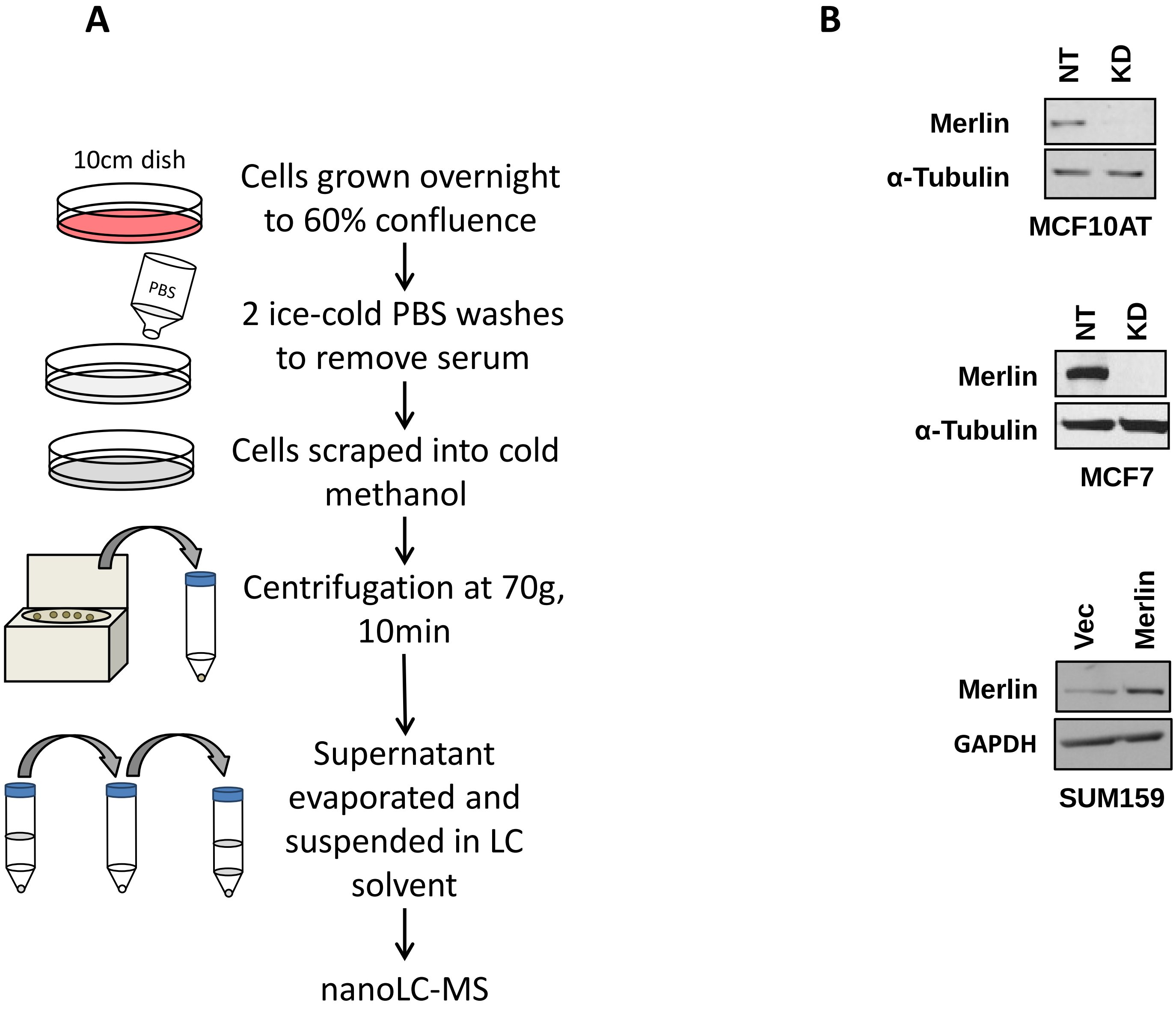

Supplementary Figure 2

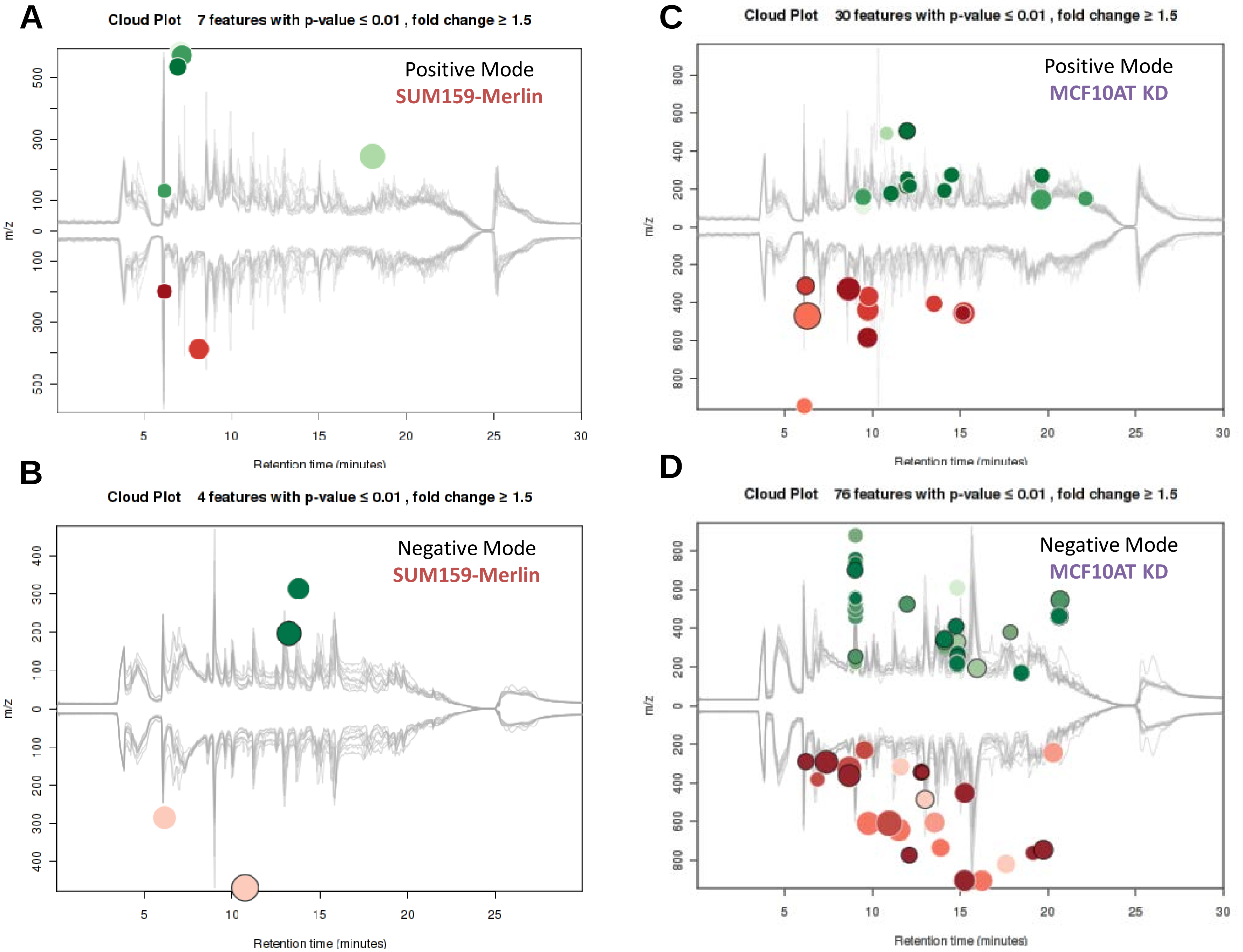

Supplementary Figure 3

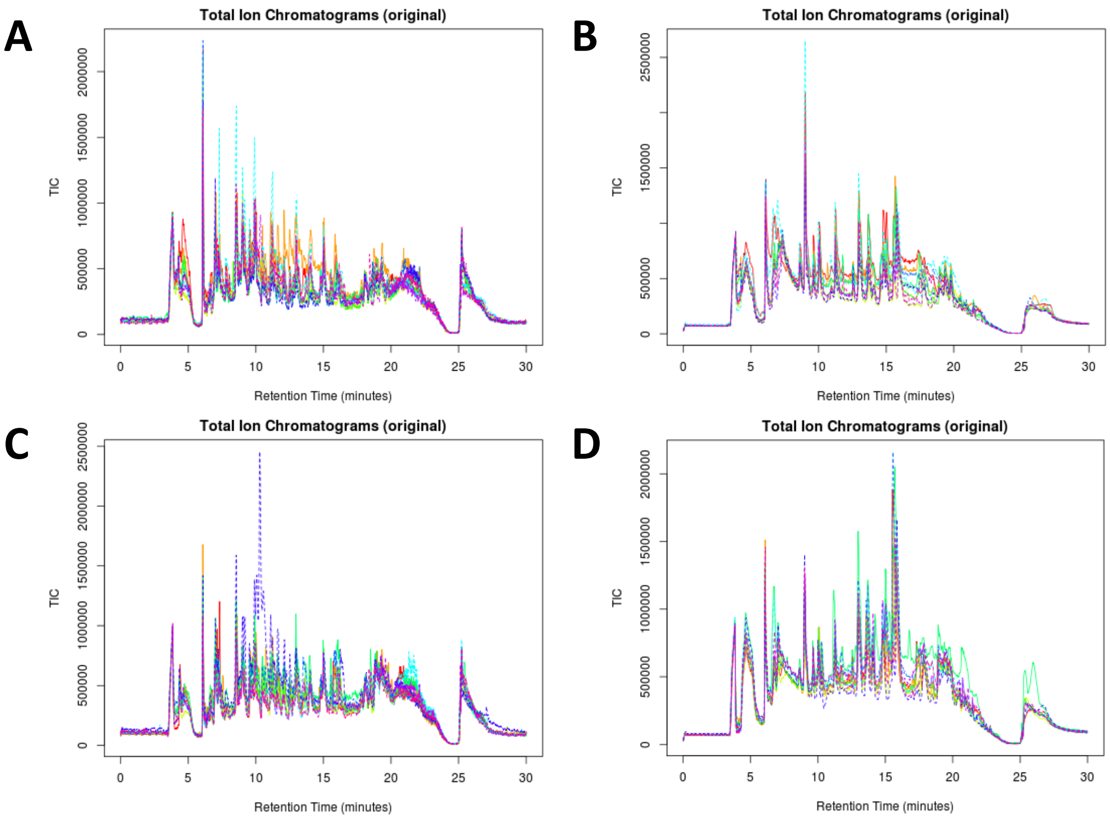

Supplementary Figure 4

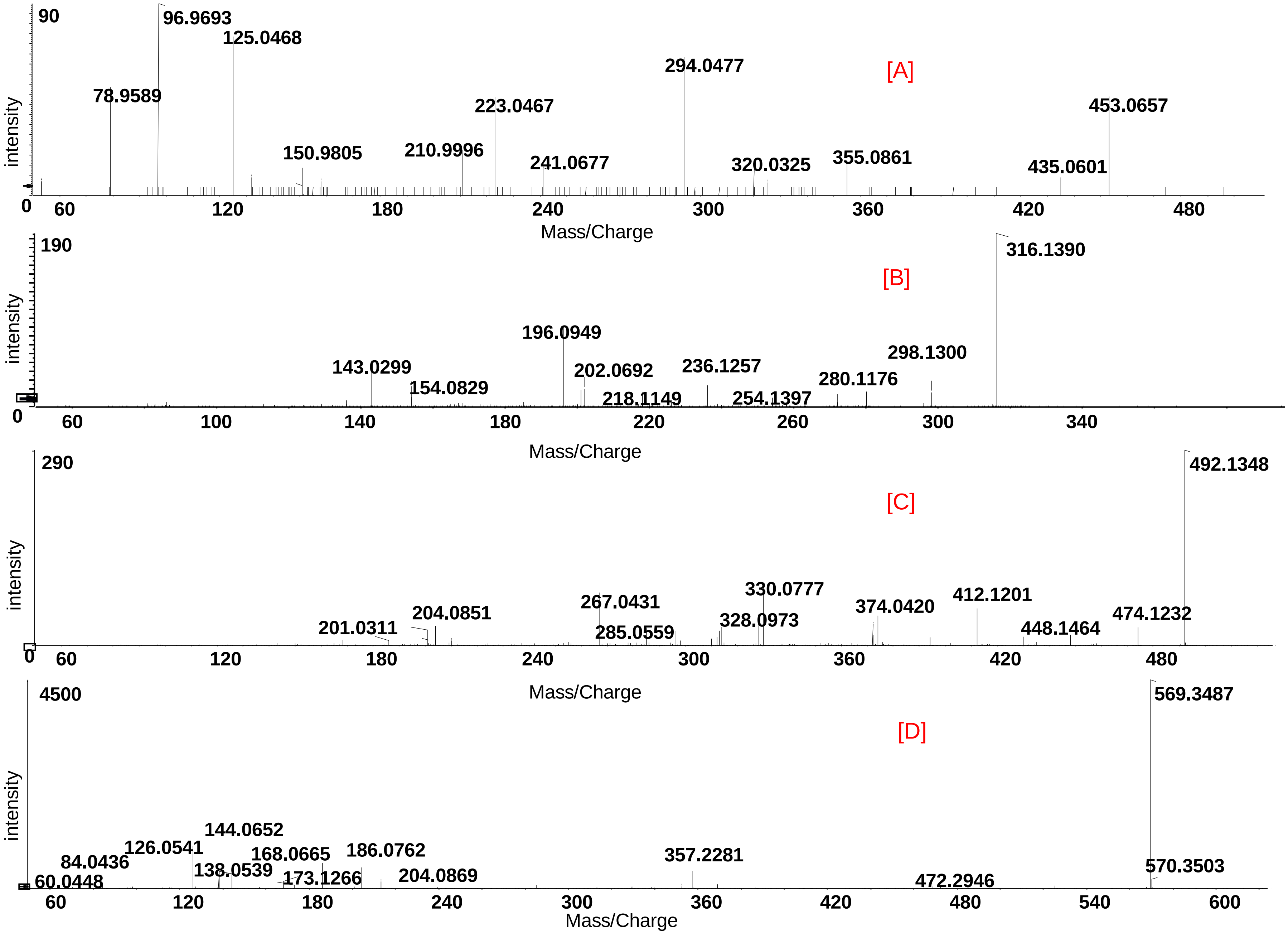

Supplementary Figure 5

A

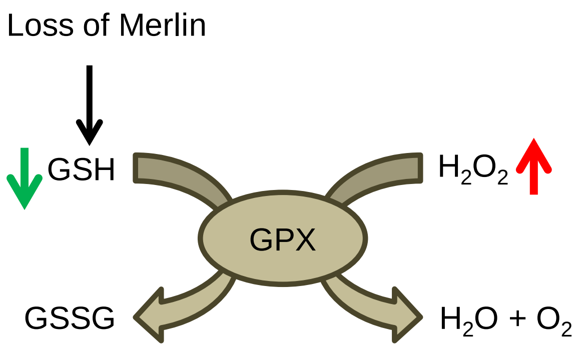

Cells	ROS levels
MCF10AT NT	100 ± 0.7
MCF10AT KD	105 ± 0.4
MCF7 NT	100 ± 0.9
MCF7 KD	103 ± 0.9

B

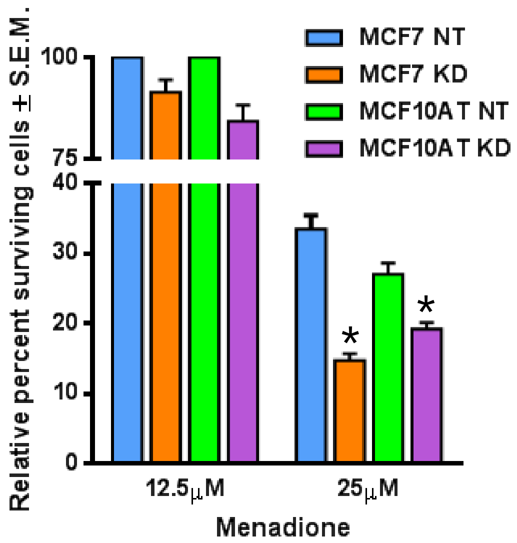

Supplementary Figure 6

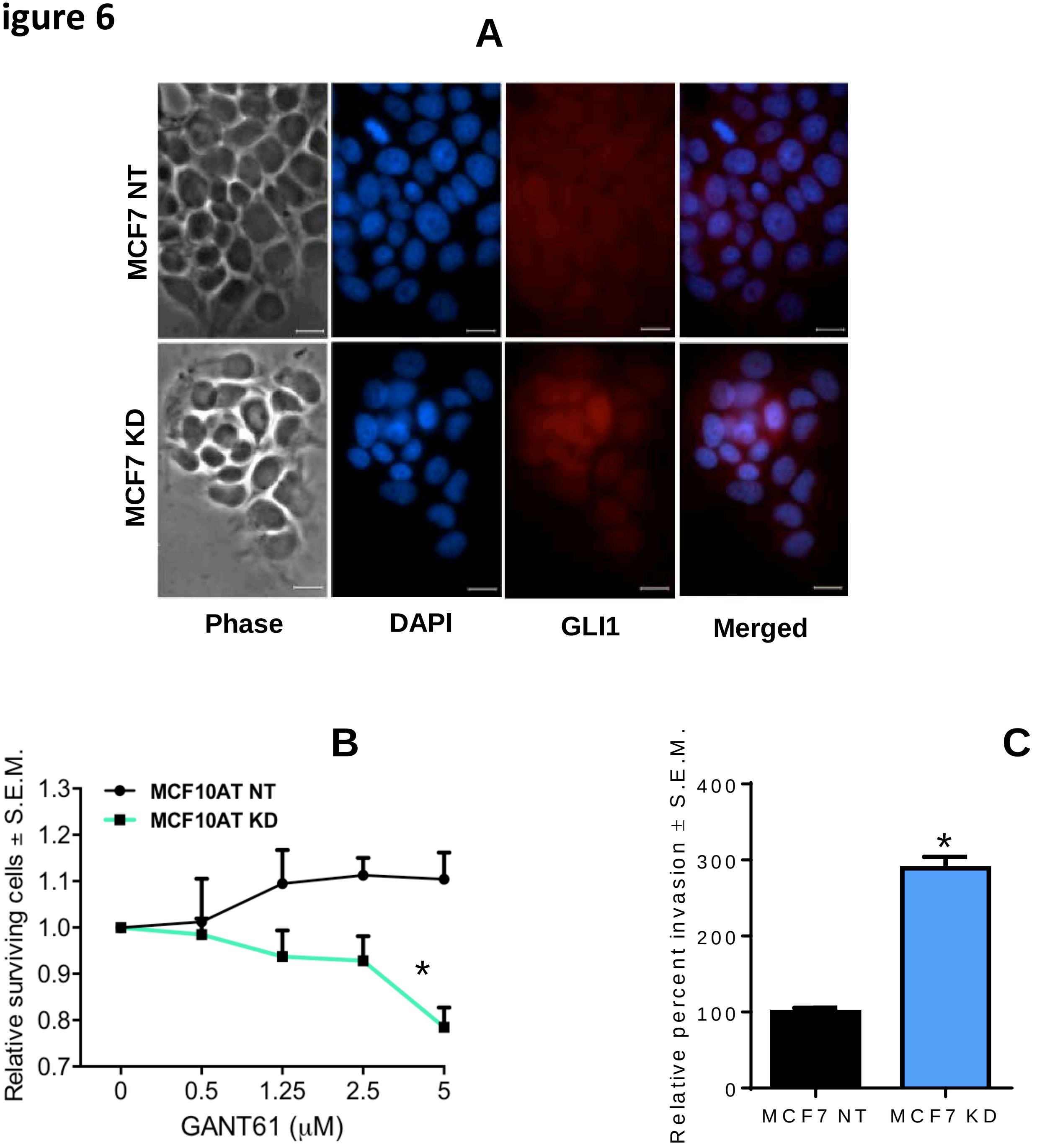

Supplementary Figure 7

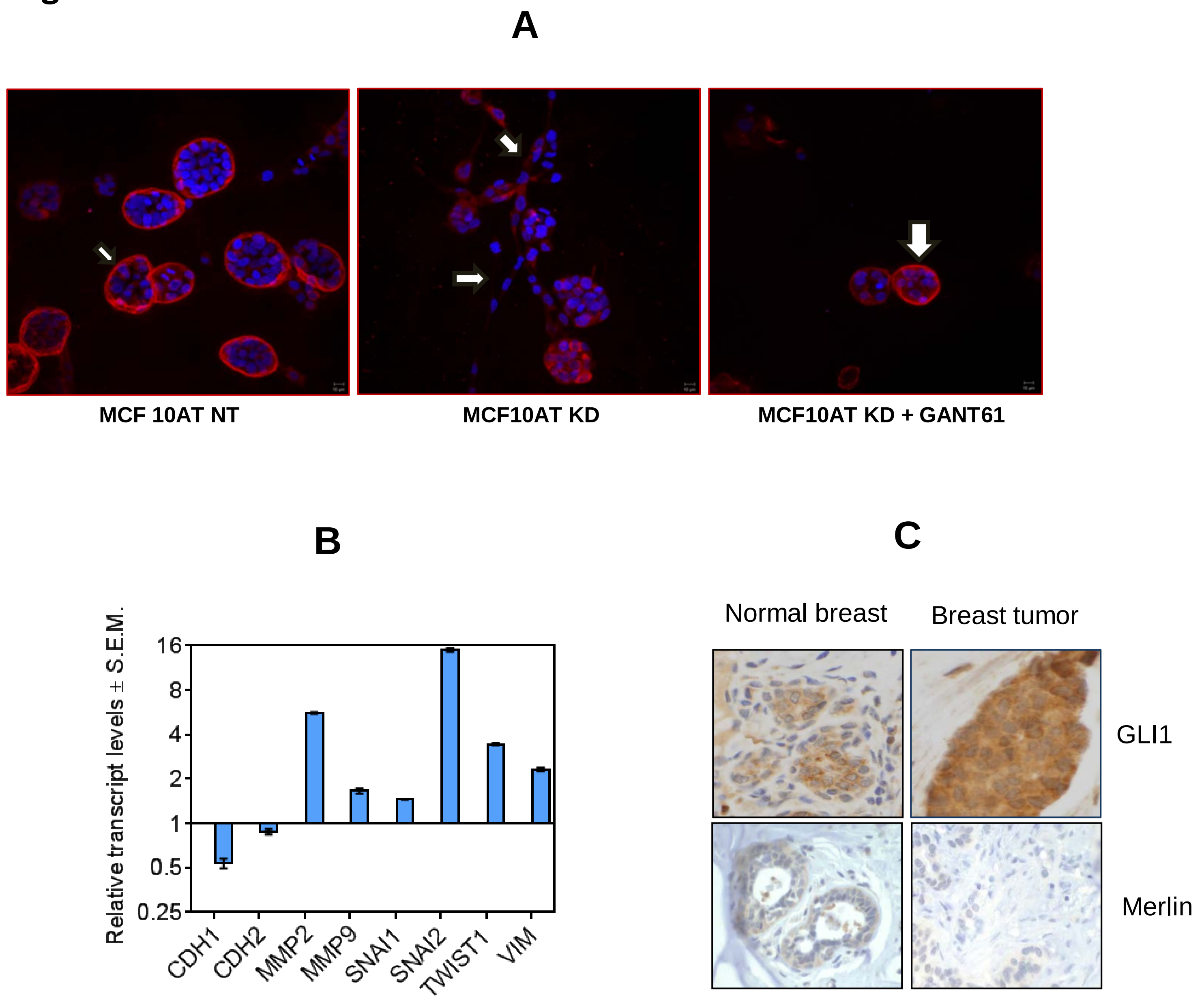

Supplementary Table 1

System	ESI	Detected Peaks	Technical Filters	FC>2	% Real Peaks Changing /Filtered Peaks
SUM159 Merlin	Positive	2792	1221	**3**	0.25 %
SUM159 Merlin	Negative	4059	1785	**4**	0.22 %
MCF10AT shMerlin	Positive	2599	1255	**14**	1.12 %
MCF10AT shMerlin	Negative	3955	1639	**26**	1.59 %
**TOTAL**		**13405**		**47**	**0.80 %**

Supplementary Table 2

A SUM159 Merlin

Pathway	Potential Matches	Pathway Metabolites	p-value	ESI mode
Glutathione Metabolism	3	9	0.00421	Positive
Alanine and Aspartate Metabolism	3	11	0.0043	Positive
Urea cycle/amino group Metabolism	3	29	0.00587	Positive
Beta-Alanine metabolism	2	7	0.00595	Positive
Glutatmate metabolism	2	7	0.00595	Positive
Tryptophan metabolism	3	38	0.00719	Positive
Vitamin B9 (folate) metabolism	2	12	0.00764	Positive
Histidine metabolism	2	12	0.00764	Positive
Arginine and Proline Metabolism	2	16	0.0092	Positive
Purine metabolism	2	17	0.00962	Positive
Tyrosine metabolism	3	58	0.0117	Positive
Pyrimidine metabolism	2	24	0.0129	Positive
Glycine, serine, alanine, and threonine metabolism	2	24	0.0129	Positive
Aspartate and asparagine metabolism	2	27	0.01448	Positive

B MCF10AT KD

Pathway	Potential Matches	Pathway Metabolites	p-value	ESI mode
Beta-Alanine metabolism	2	5	0.04348	Positive
Arginine and Proline Metabolism	2	17	0.10366	Positive
Methionine and cysteine metabolism	2	25	0.16133	Positive
Tyrosine metabolism	3	61	0.16485	Positive
Aspartate and asparagine metabolism	2	28	0.18611	Positive
Vitamin B5 - CoA biosynthesis from pantothenate	2	10	0.00561	Negative
Selenoamino acid metabolism	2	13	0.01029	Negative
Ascorbate (Vitamin C) and Aldarate Metabolism	2	16	0.01713	Negative
Androgen and estrogen biosythesis and metabolism	4	51	0.02151	Negative
Typtophan metabolism	5	38	0.02374	Negative
Drug metabolism - cytochrome P450	3	41	0.03874	Negative
Glycerophospholipid metabolism	2	23	0.0422	Negative
Sialic acid metabolism	2	23	0.0422	Negative

Supplementary Table 3

A

ESI Positive Mode

Peak	m/zmed	rtmed	log2fold	pvalue
9	386.7123	8.11	1.128	0.041
180	163.1095	20.95	1.025	0.048
23	243.2872	18.05	-1.987	0.004

B

ESI Negative Mode

Peak	m/zmed	rtmed	log2fold	pvalue
61	230.5284	7.94	2.449	0.038
39	546.6343	9.21	1.422	0.012
105	453.0613	7.14	1.249	0.038
167	571.1988	14.08	1.121	0.045

Supplementary Table 4

A

ESI Positive Mode

Peak	m/zmed	rtmed	log2fold	pvalue
3	328.1371	8.62	1.736	0.000
53	536.0890	6.25	1.589	0.007
46	492.1217	6.27	1.578	0.005
48	316.1273	7.27	1.527	0.004
21	454.7086	15.20	1.511	0.004
22	455.7097	15.16	1.277	0.004
55	261.0355	13.22	1.260	0.030
15	367.1331	9.76	1.135	0.009
72	193.5687	6.71	1.071	0.047
100	290.0779	6.12	1.013	0.045
4	175.1866	11.04	-1.001	0.003
20	157.0830	9.44	-1.050	0.016
18	144.1355	19.56	-1.250	0.023
62	171.1540	17.20	-1.755	0.015

B

ESI Negative Mode

Peak	m/zmed	rtmed	log2fold	pvalue
61	610.1838	10.93	2.215	0.003
201	468.1205	6.25	2.144	0.025
47	326.1218	8.65	1.932	0.001
73	610.1817	9.74	1.787	0.004
86	644.1649	11.49	1.718	0.006
7	292.1276	7.35	1.638	0.000
35	362.0948	8.65	1.526	0.001
16	906.3868	15.23	1.493	0.000
70	907.377	16.23	1.400	0.003
89	244.1371	20.28	1.337	0.006
218	417.1031	9.31	1.321	0.031
117	821.3387	17.59	1.160	0.008
54	230.0043	9.51	1.136	0.004
31	747.3897	19.73	1.109	0.003
111	384.0821	8.65	1.091	0.007
115	317.1536	11.58	1.085	0.012
259	417.1541	19.51	1.076	0.036
18	219.0309	14.81	-1.024	0.000
57	335.09	14.08	-1.032	0.004
92	208.3527	14.80	-1.062	0.013
8	209.0021	14.81	-1.072	0.000
63	547.2621	20.68	-1.081	0.007
30	464.1633	20.62	-1.082	0.003
38	463.1599	20.64	-1.104	0.005
240	881.3251	20.55	-1.108	0.048
98	194.0443	15.94	-1.125	0.015

Supplementary Table 5

Cell Line	ESI	Peak	m/zmed	rtmed	fold	log2fold	P-value
MCF10AT	Positive	18	144.1355	19.56	0.420	-1.249	0.023281
MCF10AT	Positive	20	157.0830	9.44	0.482	-1.050	0.016084
SUM159	Positive	180	163.1095	20.95	2.035	1.025	0.04832
MCF10AT	Positive	62	171.1540	17.20	0.296	-1.755	0.015015
MCF10AT	Positive	4	175.1866	11.04	0.499	-1.000	0.002779
MCF10AT	Positive	72	193.5687	6.71	2.100	1.070	0.047322
SUM159	Positive	23	243.2872	18.05	0.252	-1.986	0.004225
MCF10AT	Positive	55	261.0355	13.22	2.395	1.260	0.029727
MCF10AT	Positive	100	290.0779	6.12	2.017	1.012	0.045476
MCF10AT	Positive	48	316.1273	7.27	2.881	1.526	0.003921
MCF10AT	Positive	3	328.1371	8.62	3.330	1.735	0.000252
MCF10AT	Positive	15	367.1331	9.76	2.195	1.134	0.00866
SUM159	Positive	9	386.7123	8.11	2.185	1.128	0.040818
MCF10AT	Positive	21	454.7086	15.20	2.850	1.511	0.00416
MCF10AT	Positive	22	455.7097	15.16	2.422	1.276	0.003666
MCF10AT	Positive	46	492.1217	6.27	2.984	1.577	0.004579
MCF10AT	Positive	53	536.0890	6.25	3.007	1.588	0.006851

Direct Correlation with Merlin (n=11)	Inverse Correlation with Merlin (n=6)	MCF10AT	SUM159
		14	3

Supplementary Table 6

Cell Line	ESI	Peak	m/zmed	rtmed	fold	log2fold	P-value
MCF10AT	Negative	98	194.0442	15.94	0.458	-1.125	0.015027
MCF10AT	Negative	92	208.3526	14.80	0.478	-1.062	0.012705
MCF10AT	Negative	8	209.0020	14.81	0.475	-1.071	0.000421
MCF10AT	Negative	18	219.0309	14.81	0.491	-1.023	3.24E-05
MCF10AT	Negative	54	230.0042	9.51	2.197	1.136	0.00442
SUM159	Negative	61	230.5284	7.94	5.461	2.449	0.038093
MCF10AT	Negative	89	244.1371	20.28	2.526	1.337	0.006272
MCF10AT	Negative	7	292.1275	7.35	3.112	1.638	0.000252
MCF10AT	Negative	115	317.1536	11.58	2.120	1.084	0.012233
MCF10AT	Negative	47	326.1217	8.65	3.815	1.932	0.001481
MCF10AT	Negative	57	335.0899	14.08	0.488	-1.032	0.003658
MCF10AT	Negative	35	362.0947	8.65	2.880	1.526	0.001497
MCF10AT	Negative	111	384.0820	8.65	2.130	1.091	0.007017
MCF10AT	Negative	218	417.1031	9.31	2.497	1.320	0.031106
MCF10AT	Negative	259	417.1540	19.51	2.107	1.075	0.036132
SUM159	Negative	105	453.0612	7.14	2.376	1.248	0.038348
MCF10AT	Negative	38	463.1598	20.64	0.465	-1.103	0.004592
MCF10AT	Negative	30	464.1633	20.62	0.472	-1.081	0.003294
MCF10AT	Negative	201	468.1205	6.25	4.418	2.143	0.025121
SUM159	Negative	39	546.6343	9.21	2.680	1.422	0.012237
MCF10AT	Negative	63	547.2620	20.68	0.472	-1.0809	0.007435
SUM159	Negative	167	571.1988	14.08	2.174	1.120	0.045144
MCF10AT	Negative	73	610.1816	9.74	3.450	1.786	0.004041
MCF10AT	Negative	61	610.1837	10.93	4.642	2.215	0.003151
MCF10AT	Negative	86	644.1648	11.49	3.289	1.718	0.005783
MCF10AT	Negative	31	747.3896	19.73	2.157	1.109	0.003138
MCF10AT	Negative	117	821.3386	17.59	2.234	1.159	0.007854
MCF10AT	Negative	240	881.3251	20.55	0.463	-1.108	0.047707
MCF10AT	Negative	16	906.3868	15.23	2.815	1.493	0.000477
MCF10AT	Negative	70	907.3769	16.23	2.639	1.400	0.003419

		MCF10AT	SUM159
**Direct Correlation with Merlin (n=17)**	**Inverse Correlation with Merlin (n=13)**	**26**	**4**

Original Films/Images for Figure 3C showing the cropped regions in red

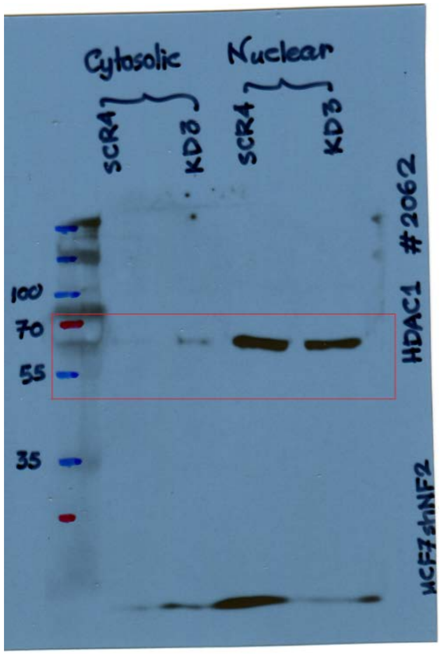

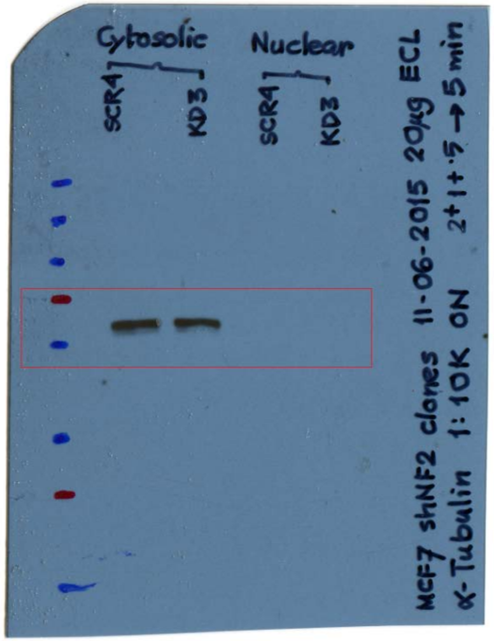

Original Films/Images for Figure 3C showing the cropped regions in red

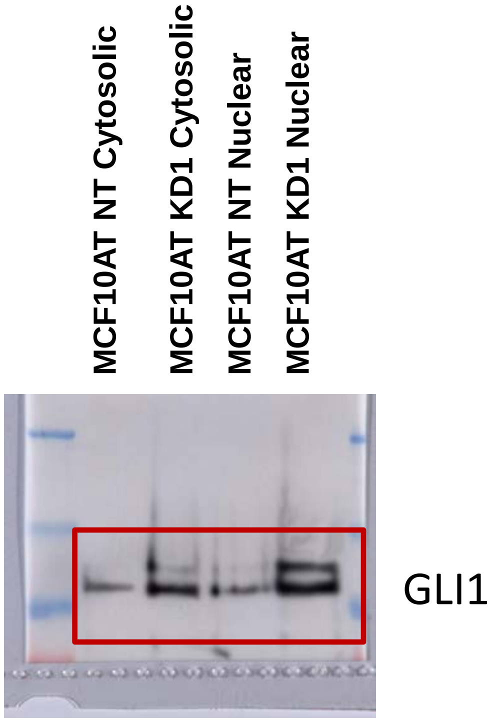

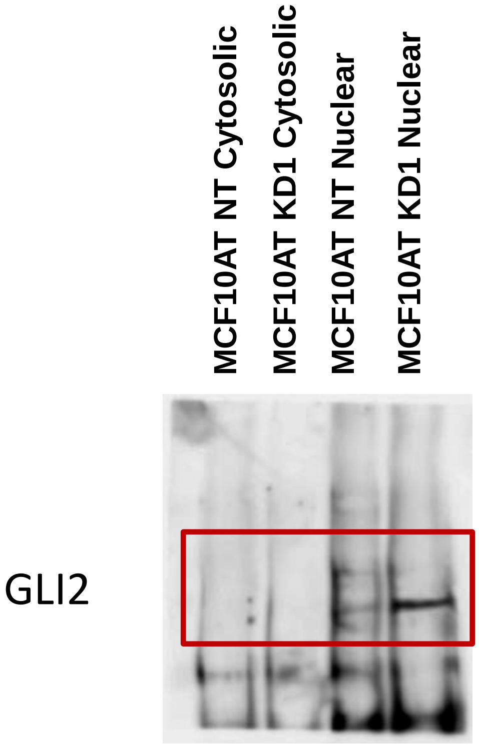

Supplement: Supplementary Information [file srep40773-s1.pdf]
